# *Fagopyrum tataricum* (Buckwheat) Improved High-Glucose-Induced Insulin Resistance in Mouse Hepatocytes and Diabetes in Fructose-Rich Diet-Induced Mice

**DOI:** 10.1155/2012/375673

**Published:** 2012-04-04

**Authors:** Chia-Chen Lee, Wei-Hsuan Hsu, Siou-Ru Shen, Yu-Hsiang Cheng, She-Ching Wu

**Affiliations:** Department of Food Science, National Chiayi University, Chiayi City 60004, Taiwan

## Abstract

*Fagopyrum tataricum* (buckwheat) is used for the treatment of type 2 diabetes mellitus in Taiwan. This study was to evaluate the antihyperglycemic and anti-insulin resistance effects of 75% ethanol extracts of buckwheat (EEB) in FL83B hepatocytes by high-glucose (33 mM) induction and in C57BL/6 mice by fructose-rich diet (FRD; 60%) induction. The active compounds of EEB (100 **μ**g/mL; 50 mg/kg bw), quercetin (6 **μ**g/mL; 3 mg/kg bw), and rutin (23 **μ**g/mL; 11.5 mg/kg bw) were also employed to treat FL83B hepatocytes and animal. Results indicated that EEB, rutin, and quercetin + rutin significantly improved 2-NBDG uptake via promoting Akt phosphorylation and preventing PPAR**γ** degradation caused by high-glucose induction for 48 h in FL83B hepatocytes. We also found that EEB could elevate hepatic antioxidant enzymes activities to attenuate insulin resistance as well as its antioxidation caused by rutin and quercetin. Finally, EEB also inhibited increases in blood glucose and insulin levels of C57BL/6 mice induced by FRD.

## 1. Introduction

Type 2 diabetes mellitus (T2DM) is a chronic disease caused by deficient insulin secretion or ineffective insulin activity, which negatively affects carbohydrate metabolism. Medicinal plants are used as a common alternative treatment for T2DM in many parts of the world. Insulin resistance is associated with inflammatory factors such as tumor necrosis factor-*α* (TNF-*α*) and interleukin-6 (IL-6) in T2DM patients. Cellular stress due to obesity is thought to be associated with the disturbance of homeostasis in the endoplasmic reticulum (ER). Hepatic regulation of glucose homeostasis is the major factor controlling plasma glucose concentrations, and the induction of hepatic ER stress and oxidative stress resulting in insulin resistance has been investigated [1].

High-fructose diet upregulates hepatic expression of the sterol regulatory element binding protein-1c (SREBP-1c), a key transcription factor for hepatic expression of lipogenic enzymes, but down regulates the expression of PPAR*α* (promoting fatty acid oxidation) [2, 3]. The study also investigates the fructose-inducing effect in C57BL/6 mice and has found that fructose would promote SREBP-1c promoter activity resulting in hepatic lipogenesis [4]. Moreover, fructose is employed to induce insulin resistance, hepatic steatosis, and the metabolic syndrome [5]. Fructose is a highly lipogenic sugar that has profound metabolic effects in the liver resulting in metabolic syndrome, and fructose does not stimulate insulin secretion [6]. The rate of hepatic uptake of fructose from portal circulation is greater than the rate of glucose uptake, and because fructose metabolism bypasses phosphofructokinase, fructose metabolism is not under the regulatory control of insulin [7]. On the other hand, fructose may activate SREBP-1c which activates genes involved in de novo lipogenesis, and triglyceride [8].

*Fagopyrum tataricum* (buckwheat) is a herbaceous plant that belongs to the Polygonaceae family. It has now been introduced in many countries, because the seeds of this herb are a healthy and nutritionally important food item. Rutin has been found to be the major ingredient of buckwheat [9]. Tartary buckwheat (*F. tataricum*) contains more rutin and quercetin than common buckwheat (*F. esculentum*) dose; rutin is known to have antioxidative activity [10]. Due to the rise in recent years to investigate the anti-diabetic activity of antioxidants, anti-insulin resistance of rutin, quercetin, and the 75% ethanol extracts from buckwheat (EEB) *in vivo* and *in vitro* were investigated in this study.

## 2. Materials and Methods

### 2.1. Materials and Chemicals

3-(4,5-Dimethylthiazol-2-yl)-2,5-diphenyltetrazolium bromide (MTT), glucose, quercetin, Triton-X 100, rutin, and trypsin were purchased from Sigma Co. (St. Louis, MO, USA). Sodium bicarbonate, fetal bovine serum (FBS), F12-K medium, penicillin, and streptomycin were purchased from HyClone Laboratories (Logan, UT, USA). The Bio-Rad protein assay dye was from Bio-Rad Laboratories (Hercules, CA, USA). 2-[N-(7-Nitrobenz-2-oxa-1,3-diazol-4-yl)amino]-2-deoxy-d-glucose (2-NBDG) was from Invitrogen (Carlsbad, CA, USA).

### 2.2. Preparation of Sample

The seeds of *F. tataricum* (buckwheat) were provided by Taiwan Golden Buckwheat Limited company) and then were freeze-dried and ground. Approximately 2.5 kg of the buckwheat powder was extracted by 25 L of 75% ethanol for 2 days. After extraction, the ethanol extracts were vacuum-concentrated and freeze-dried. The extract powder was stored at −20°C until used. The 75% ethanol extracts of buckwheat was shown as EEB in this study.

### 2.3. Cell Culture

 FL83B cells were seeded in 10 cm dishes at a density of 5 × 10^5^ per well and grown until 80% confluence was reached. Subsequently, insulin resistance was induced in these cells and glucose uptake was determined [11]. FL83B cell line is a mouse normal liver cell from the Bioresource Collection and Research Center (BCRC) in Taiwan (Hsinchu, Taiwan), which is cultured in the F12-K medium supplemented with 10% heat-inactivated FBS and antibiotics (100 unit/mL penicillin and 100 *μ*g/mL streptomycin). Cells were cultured at 37°C in a humidified atmosphere of 5% CO_2_.

### 2.4. High-Performance Liquid Chromatography (HPLC) Assay

**Figure 1:**
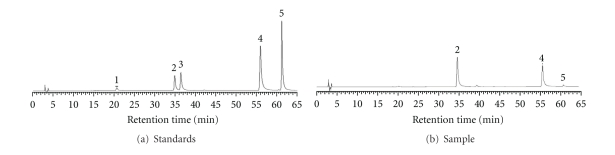
(a) The HPLC chromatogram of standards (rutin and quercetin): (1) caffic acid, (2) rutin, (3) quercetin-3-glucoside, (4) quercetin, and (5) kaempferol. (b) The HPLC chromatogram of sample (75% ethanol extracts of *F. tataricum*).

HPLC was performed with a Hitachi liquid chromatograph (Hitachi, Ltd., Tokyo, Japan) consisting of a model L-6200 pump and a model L-4200 UV-Vis detector set at 320 nm. The analyses were carried out on a LiChrospher RP-18 column (250 mm*·*4.6 mm i.d., 5 *μ*m, E. Merck Co., Darmstadt, Germany). Extracts were filtered through a 0.45 *μ*m filter before use. The mobile phase A was 2% acetic acid, and the mobile phase B was 0.5% acetic acid/water (1 : 1; v/v). Caffic acid, rutin, quercetin, kaempferol, and quercetin-3-glucoside were determined by ultraviolet detector (Hitachi L-7455 diode array detector). Caffic acid, rutin, quercetin, kaempferol, and quercetin-3-glucoside were identified by comparison of their retention time (Rt) values and UV spectra with those of known standards and determined by peak areas from the chromatograms [12]. Results suggested that 228.8 mg/g of rutin and 58.6 mg/g of quercetin were contained in EEB (Figure 1).

### 2.5. Cell Viability

Mouse FL83B cells (1.5 × 10^5^ cells per well) were seeded into 24-well plates overnight. Cells were treated with high glucose (33 mM) and sample (quercetin/rutin/EEB) in free-serum F12-K medium for 48 h. Subsequently, cells were washed with phosphate buffered saline (PBS) twice, and the supernatants were exchanged with 1 mL of medium and MTT (0.5 mg/mL) to react for 2 h at 37°C. After reaction, removing medium, and washing cells with PBS, the MTT reacted product (formazan crystals) was dissolved with 0.5 mL of dimethyl sulfoxide (DMSO), and the absorption was measured at 570 nm by an ELISA reader for cell viability assay.

### 2.6. Insulin Resistance Induction and Glucose (2-NBDG) Uptake

Glucose uptake of FL83B cells was assessed using the fluorescent glucose analog, 2-NBDG. Briefly, cells were treated with high glucose (33 mM) and sample in serum-free medium for 48 hours, and then the medium was replaced with Krebs-Ringer-Bicarbonate (KRB) buffer containing insulin (500 nM; final concentration) and 2-NBDG (160 *μ*M; final concentration) for 20 min for incubation at 37°C. Free 2-NBDG was washed out from cultures after treatment and measured 2-NBDG with a FACS flow cytometer (BD Biosciences, San Jose, CA, USA) and analyzed using CellQuest software [11].

### 2.7. Western Blot Analysis

FL83B cells were lysed in ice-cold lysis buffer containing 20 mM of Tris-HCl (pH 7.4), 1% of Triton X-100, 0.1% of SDS, 2 mM of EDTA, 10 mM of NaF, 1 mM of phenylmethylsulfonyl fluoride (PMSF), 500 *μ*M of sodium vanadate, and 10 *μ*g/mL of aprotinin overnight. And then the cell lysates were sonicated with ice cooling (four times each 15 s) and then centrifuged (12,000 ×g, 10 min) to recover the supernatant. The supernatant was taken as the cell extract. The protein concentration in the cell extract was determined using a Bio-Rad protein assay kit. The samples were subjected to 10% sodium dodecyl sulfate-polyacrylamide gel electrophoresis (SDS-PAGE). The protein spots were electrotransferred to a polyvinylidene difluoride (PVDF) membrane. The membrane was incubated with block buffer (PBS containing 0.05% of Tween-20 and 5% w/v nonfat dry milk) for 1 h, washed with PBS containing 0.05% Tween-20 (PBST) three times, and then probed with anti-Akt antibody, anti-PTP1B, anti-GS, anti-p-PKC, anti-PKC, anti-AMPK, anti-p-Akt, anti-GLUT2, and anti-PPAR-*γ* antibodies (Cell Signaling Technology, Beverly, MA, USA) overnight at 4°C. In addition, the intensity of the blots probed with 1 : 1000 diluted solution of mouse monoclonal antibody to bind GAPDH (Cell Signaling Technology) was used as the control to ensure that a constant amount of protein was loaded into each lane of the gel. The membrane was washed three times each for 5 min in PBST, shaken in a solution of HRP-linked anti-rabbit IgG secondary antibody, and washed three more times each for 5 min in PBST. The expressions of proteins were detected by enhanced chemiluminescent (ECL) reagent (Millipore, Billerica, MA, USA).

### 2.8. Animals Study

C57BL6 mice (4 weeks old) were obtained from BioLASCO, Taiwan Co., Ltd. in this study. Animals were provided with food and water ad libitum. Animals were subjected to 12 h light/dark cycle with a maintained relative humidity of 60% and a temperature at 25°C. The experiments were carried out in a qualified animal breeding room in the animal center at our institute. Hyperglycemia and hyperinsulinemia in mice were induced by fructose-rich diet (FRD; 60%) for 8 weeks of induction [13]. The animals were randomly divided into 6 groups (*n* = 12), including (a) control, (b) fructose-rich diet (FRD), (c) FRD + quercetin (3 mg/kg bw), (d) FRD + rutin (11.5 mg/kg bw), (e) FRD + EEB (50 mg/kg bw), and (f) FRD + rutin + quercetin. The doses of rutin and quercetin were equivalent to those administered to the EEB administration group.

### 2.9. Oral Glucose Tolerance Test (OGTT)

The OGTT was performed at week 4 and week 8. The experiment was performed on animals after fasting for 12 h (free access to water). Animals were given glucose (2 g/kg of body weight) with an oral administration. Blood samples were collected from the tail vein at times 0, 30, 60, 90, and 120 min after glucose administration. Homeostasis model assessment of insulin resistance (HOMA-IR) was calculated according to the formula HOMA-IR = fasting insulin × fasting blood glucose/22.5 [14, 15].

### 2.10. Assays for Blood Glucose

Blood glucose was immediately determined by the glucose oxidase method, using an analyzer [16].

### 2.11. Assay for Antioxidase Activity

 Glutathione peroxidase (GPx) activity was determined as previously described [17]. Glutathione reductase (GR) activity determination was according to Bellomo et al. (1987) [18]. The catalase (CAT) activity was determined by the method of Aebi (1984) [19]. SOD activity was determined by the method of S. Marklund and G. Marklund (1974) [20].

### 2.12. Assay for Hepatic and Pancreatic Reactive Oxygen Species (ROS)

The ROS levels were assayed with nitroblue tetrazolium (NBT), which is reduced to form blue-black formazan. In this assay, 100 *μ*L of homogenates reacted with 10 mg/mL of NBT and measured by the absorbance at 570 nm [21].

### 2.13. Assay for Insulin Level

Insulin was determined by the insulin kit derived from Mercodia AB (Uppsala, Sweden).

### 2.14. Histopathologic Studies

 Liver tissues were trimmed (2 mm thickness) and fixed (buffer formaldehyde). The fixed tissues were processed including those embedded in paraffin, sectioned, and rehydrated. The histological examination by the previous conventional methods evaluated the index of ethanol-induced necrosis by assessing the morphological changes in the liver sections stained with hematoxylin and eosin (H and E) [22].

### 2.15. Statistical Analysis

Experimental results were averaged triplicate analysis. The data were recorded as mean ± standard deviation (SD) and analysis by statistical analysis system (SAS Inc., Cary, NC, USA). One-way analysis of variance was performed by ANOVA procedures. Significant differences between means were determined by Duncan's multiple range tests. Results were considered statistically significant at *P* < 0.05.

## 3. Results and Discussion

### 3.1. Effects of EEB, Rutin, and Quercetin on High-Glucose-Induced Insulin Resistance in the FL83B Hepatocytes for 48 h

**Figure 2:**
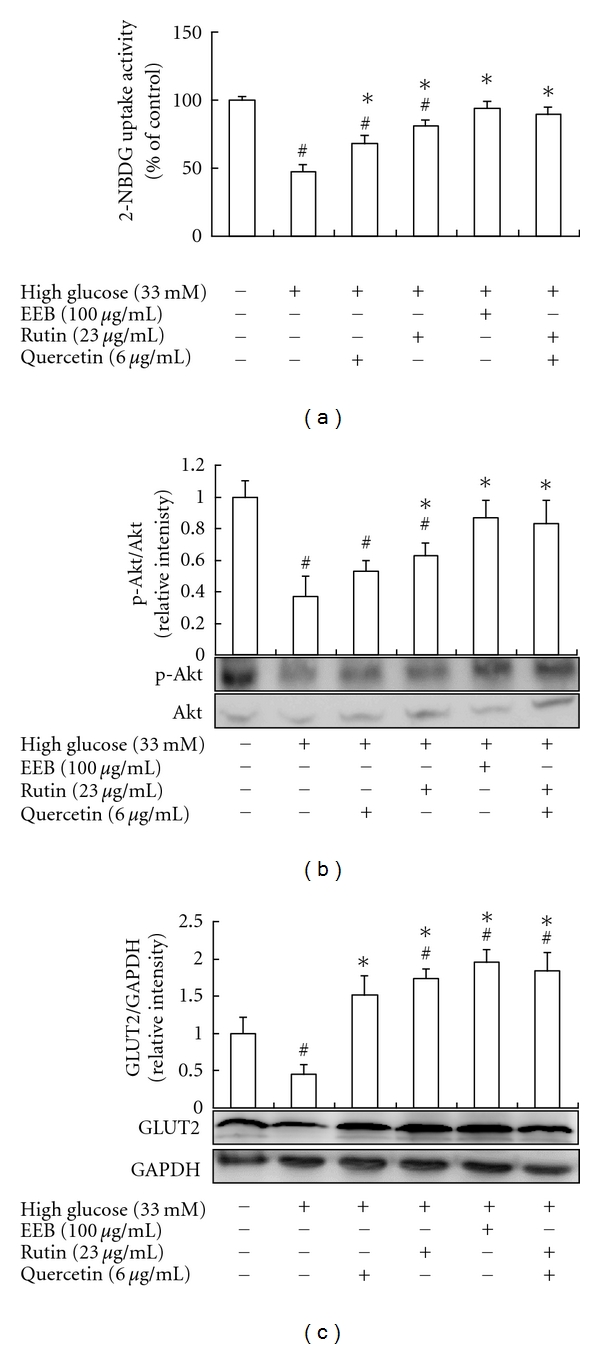
Effects of EEB on 2-NBDG uptake (a), Akt phosphorylation (b), and GLUT2 expression (c) of FL83B hepatocytes induced by high glucose. FL83B cells were incubated in serum-free F12K medium with glucose (33 mM; final concentration) with or without EEB, quercetin, rutin, and quercetin + rutin for 48 h. EEB: 75% ethanol extracts of buckwheat. ^#^Significantly different (*P* < 0.05) from normal; *significantly different (*P* < 0.05) from high-glucose treating group.

Caffic acid, rutin, quercetin, kaempferol, and quercetin-3-glucoside of EEB were identified by HPLC. Results suggested that 228.8 mg/g of rutin and 58.6 mg/g of quercetin were contained in EEB (Figure 1). We performed a 2-NBDG uptake test involving FL83B cells with high-glucose (33 mM)-induced insulin resistance to evaluate the effects of EEB (100 *μ*g/mL), rutin (23 *μ*g/mL; 37 *μ*M), and quercetin (6 *μ*g/mL; 20 *μ*M) on improving insulin sensitivity. The results showed that EEB, quercetin, and rutin significantly increased glucose uptake in these cells (Figure 2(a)). Furthermore, the treating concentrations of EEB, rutin, and quercetin without cytotoxic effects were found in FL83B hepatocytes (data not shown).

Akt is a Ser/Thr protein kinase that plays a key role in the translocation of glucose transporter (GLUT) to the plasma membrane via a signal transduction cascade involving insulin treatment [23]. We determined whether activated Akt was involved in the anti-insulin resistance effect of EEB, rutin, quercetin, and rutin + quercetin on glucose uptake. The treatment of high glucose significantly inhibited Akt phosphorylation in FL83B hepatocytes (Figure 2(b)). These results showed that exposure to high concentrations of glucose induces an insulin resistance-like condition including inhibition of the Akt pathway and EEB, rutin, quercetin, and rutin + quercetin could overcome the insulin resistance by activating the Akt pathways, thus resulting in increased glucose uptake.

GLUT2 is the major glucose transporter expressed in hepatocytes, insulin-secreting pancreatic *β*-cells and absorptive epithelial cells of the intestinal mucosa and kidney. GLUT2 is thought to act as a glucose-sensing apparatus that plays a role in blood glucose homeostasis, by responding to changes in blood glucose concentration and altering the rate of glucose uptake by hepatocytes. High-glucose levels decreased GLUT2 protein expression in FL83B cells, but EEB, rutin, quercetin, and rutin + quercetin markedly increased GLUT2 protein expression (Figure 2(c)). Results showed that EEB, rutin, and quercetin promoted Akt phosphorylation, in turn promoting GLUT2 translocation into plasma membrane of FL83B cells thereby increasing glucose uptake and alleviating insulin resistance induced by high-glucose. Although rutin + quercetin treatment did not show the synthetic effect on 2-NBDG uptake, Akt phosphorylation, and GLUT2 expression in high-glucose-induced FL83B hepatocytes compared to quercetin- or rutin-treated groups.

### 3.2. Effects of EEB, Rutin, and Quercetin on AMP-Dependent Protein Kinase (AMPK), Protein Tyrosine Phosphatase 1B (PTP1B), and Glycogen Synthase (GS) Expressions in FL83B Hepatocytes

AMPK is a conserved intracellular energy sensor that plays a central role in the regulation of glucose and lipid metabolism, and AMPK has multiple biological effects, including the regulation of intracellular glucose transport [24]. Recent investigations suggest that AMPK could potentially be beneficial as a therapeutic target in the treatment of diabetes and obesity [25]. However, AMPK expression would be inhibited by oxidative stress and ER stress in inflammatory factors or high-glucose induction downregulating AMPK expression and phosphorylation [1, 5, 6, 8]. On the other hand, hepatic specific PTP1B plays a pivotal role in glucose and lipid metabolism. Inhibition of PTP1B in the peripheral tissues may be beneficial with respect to the treatment of diabetes as well as the treatment of metabolic syndrome and reduction of cardiovascular risk. Study has demonstrated that PTP1B expression involved in ER stress in high-glucose induction [1, 24]. In addition, the natural product, monascin, identified from *Monascus*-fermented products has been demonstrated to show the inhibitory activity for PTP1B expression in insulin-resistance-induced C2C12 cells [26].

**Figure 3:**
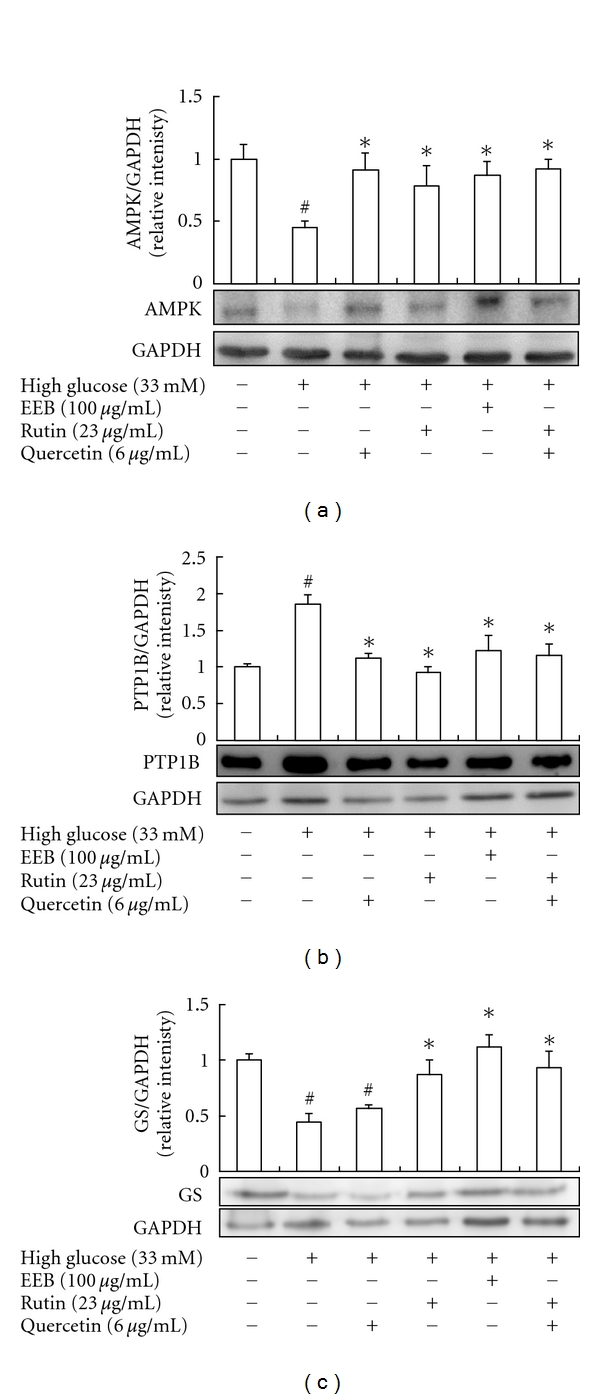
Effects of EEB on AMPK (a), PTP1B (b), and GS expressions (c) of FL83B hepatocytes induced by high glucose. FL83B cells were incubated in serum-free F12K medium with glucose (33 mM; final concentration) with or without EEB, quercetin, rutin, and quercetin + rutin for 48 h. EEB: 75% ethanol extracts of buckwheat. ^#^Significantly different (*P* < 0.05) from normal; *significantly different (*P* < 0.05) from high-glucose-treated group.

We evaluated the effects of EEB, rutin, and quercetin on AMPK and PTP1B expression of FL83B hepatocytes induced by high-glucose treatment for 48 h. Results indicated that EEB, rutin, and quercetin significantly prevented a decrease in AMPK (Figure 3(a)), and the inhibition of PTP1B was found in EEB, rutin, and quercetin treatments in high-glucose-induced FL83B hepatocytes (Figure 3(b)). These results showed that EEB, rutin, and quercetin could significantly regulate AMPK and PTP1B activity in FL83B hepatocytes thereby attenuating insulin resistance and promoting 2-NBDG uptake.

Moreover, we investigated the GS expression of FL83B hepatocytes induced by high-glucose treatment for 48 h. Results indicated that EEB, rutin, and rutin + quercetin could promote GS expression compared to the high-glucose-induced group; however, this effect was not found in the quercetin-treated group (Figure 3(c)).

### 3.3. Antioxidative Stress and Anti-PPAR*γ* Phosphorylation by EEB, Rutin, and Quercetin in FL83B Hepatocytes

**Figure 4:**
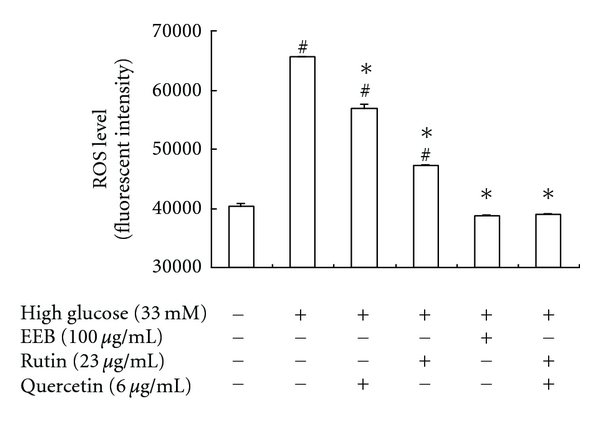
Effects of EEB on ROS level in FL83B hepatocytes induced by high glucose. FL83B cells were incubated in serum-free F12K medium with glucose (33 mM; final concentration) with or without EEB, quercetin, rutin, and quercetin + rutin for 48 h. EEB: 75% ethanol extracts of buckwheat. ^#^Significantly different (*P* < 0.05) from normal; *significantly different (*P* < 0.05) from high-glucose-treated group.

**Figure 5:**
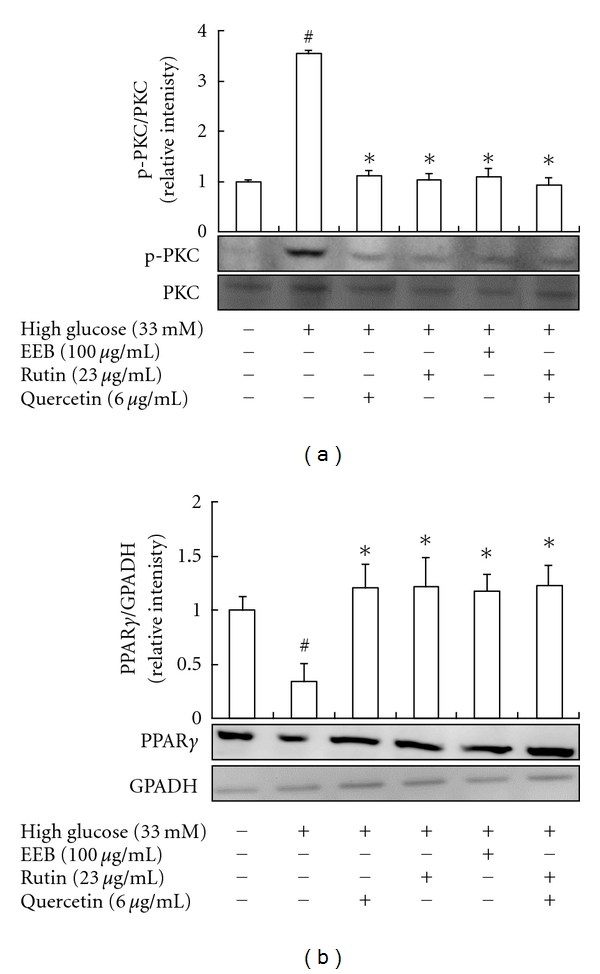
Effects of EEB on PKC phosphorylation (a) and PPAR*γ* expressions (b) of FL83B hepatocytes induced by high glucose. FL83B cells were incubated in serum-free F12K medium with glucose (33 mM; final concentration) with or without EEB, quercetin, rutin, and quercetin + rutin for 48 h. EEB: 75% ethanol extracts of buckwheat. ^#^Significantly different (*P* < 0.05) from normal; *significantly different (*P* < 0.05) from high-glucose-treated group.

High-glucose levels have been shown to induce the activities of inflammatory cytokines, chemokines, p38 mitogen-activated protein kinase, reactive oxygen species (ROS), protein kinase C (PKC), and nuclear factor-*κ*B (NF-*κ*B) activity in clinical and experimental systems [27–29]. We evaluated the inhibitory effect of EEB on ROS production in this study. High-glucose levels significantly increased ROS production, whereas EEB, rutin, quercetin, and rutin + quercetin treatments could decrease ROS production in high-glucose-induced FL83B hepatocytes (Figure 4). Activation of p-PKC directly contributes to the oxidative stress and membrane-associated NADPH oxidases, which further leads to excessive ROS production. Results suggested that EEB, rutin, quercetin, and rutin + quercetin markedly inhibited PKC phosphorylation caused by high-glucose induction for 48 h (Figure 5(a)).

Peroxisome proliferator-activated receptors (PPARs) regulate cellular development and differentiation and govern cellular bioenergetics by modulating fat and glucose metabolism and inflammatory responses [30]. There are three PPAR subtypes, including PPAR-*α*, PPAR-*γ*, and PPAR-*δ*. All three subtypes can modulate DNA transcription by binding to specific peroxisome-proliferator-response elements (PPREs) on target genes. Moreover, PPAR-*γ* plays an important role in the development of insulin resistance. PPAR*γ* plays a key role in adipogenesis, survival of mature adipocytes, fatty acid uptake, lipid storage, and systemic energy homeostasis. The metabolic regulation of PPAR-*γ* for glucose homeostasis is investigated in the study [31]. High-glucose induction attenuating insulin sensitivity has been found via activating PKC [26]. PKC inhibits PPAR-*γ* function via direct phosphorylation at serine residues, affecting DNA-binding activity and increasing PPAR-*γ* degradation by the ubiquitin-proteasome-dependent pathway [26, 31].

Therefore, we postulated that the EEB, rutin, quercetin, and rutin + quercetin could prevent a decrease in PPAR-*γ* of high-glucose-induced FL83B hepatocytes; this result may attribute to EEB, rutin, quercetin, and rutin + quercetin significantly inhibiting p-PKC activation thereby attenuating PPAR*γ* phosphorylation and degradation (Figure 5(b)).

### 3.4. The Regulation of EEB, Rutin, Quercetin, and Rutin + Quercetin on Blood Glucose *In Vivo*

Fructose is employed to induce insulin resistance, hepatic steatosis, and the metabolic syndrome [32]. Fructose is a highly lipogenic sugar that has profound metabolic effects in the liver resulting in metabolic syndrome, and fructose does not stimulate insulin secretion [6]. The rate of hepatic uptake of fructose from portal circulation is greater than the rate of glucose uptake, and because fructose metabolism bypasses phosphofructokinase, fructose metabolism is not under the regulatory control of insulin [7].

**Figure 6:**
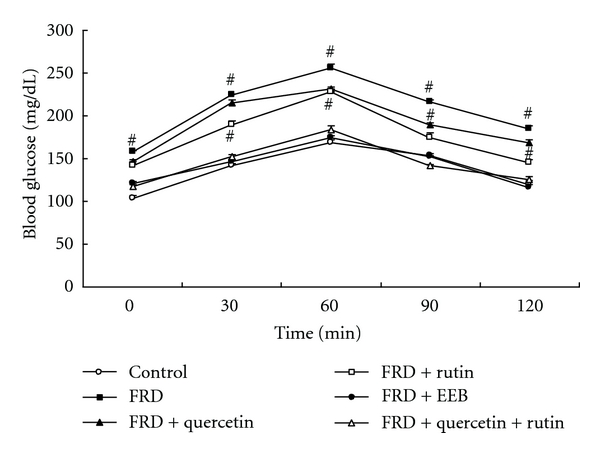
The regulatory effect of EEB, quercetin, rutin, and quercetin + rutin on OGTT in C57BL/6 mice induced by fructose-rich diet (FRD; 60%) for 8 weeks. The animals were randomly divided into 6 groups (*n* = 12), including (a) control, (b) fructose-rich diet (FRD), (c) FRD + quercetin (3 mg/kg bw), (d) FRD + rutin (11.5 mg/kg bw), (e) FRD + EEB (50 mg/kg bw), and (f) FRD + rutin + quercetin. The doses of rutin and quercetin were equivalent to those administered to the EEB administration group. EEB: 75% ethanol extracts of buckwheat. Data are presented as the mean ± SEM. ^#^Significantly different (*P* < 0.05) from control group.

The levels of blood glucose and insulin in the FRD-induced group were significantly increased compared with the normal group, suggesting that the FRD markedly induced hyperinsulinemia and hyperglycemia, and the elevations of blood glucose and insulin both were inhibited by EEB and rutin/quercetin + rutin treatments; moreover, the HOMA-IR value by FRD induction was significantly reduced by EEB and rutin/quercetin + rutin treatments (Table 1). On the other hand, the improvement of EEB and rutin/quercetin + rutin administrations for regulating blood glucose in OGTT test was significantly observed compared to the control group and FRD-induced group, suggesting that the hypoglycemic activity of rutin + quercetin and EEB are both greater than rutin or quercetin administration from 30 min to 120 min (Figure 6). However, these effects were not found in quercetin administration group, indicating that EEB and which active compound (rutin) both improved insulin sensitivity in C57BL/6 mice induced by FRD.

### 3.5. The Improvements of EEB, Rutin, Quercetin, and Rutin + Quercetin on Fatty Acid/Cholesterol Generation and Accumulation

**Figure 7:**
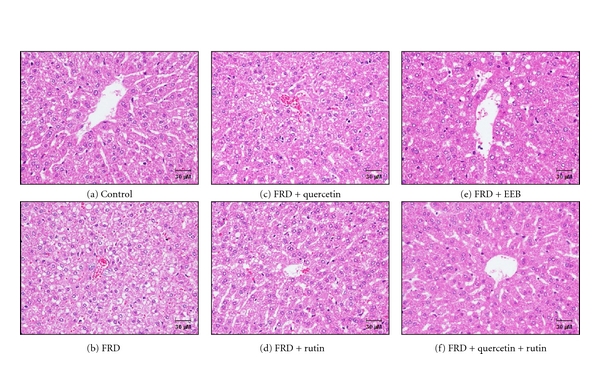
The effects of EEB, quercetin, rutin, and quercetin + rutin on fatty acid accumulation in liver of C57BL/6 mice induced by fructose-rich diet (FRD; 60%) for 8 weeks. The animals were randomly divided into 6 groups (*n* = 12), including (a) control, (b) fructose-rich diet (FRD), (c) FRD + quercetin (3 mg/kg bw), (d) FRD + rutin (11.5 mg/kg bw), (e) FRD + EEB (50 mg/kg bw), and (f) FRD + rutin + quercetin. EEB: 75% ethanol extracts of buckwheat. The doses of rutin and quercetin were equivalent to those administered to the EEB administration group.

Plasma and hepatic fatty acid/cholesterol are commonly associated with impaired insulin-mediated glucose uptake in related tissues and coexist with type 2 diabetes and obesity [33]. As shown in Table 2, EEB and rutin/quercetin + rutin could improve plasma and hepatic TC, TG, HDL-C, and LDL-C levels compared to the FRD-induced group, thereby showing antihyperglycemic and antihyperinsulinemic activities. In addition, FRD induction for 8 weeks significantly increased fatty degeneration and accumulation provided histopathological evidence of liver tissue; however, these histopathologic injuries were improved by EEB, rutin/quercetin + rutin administration (Figure 7).

### 3.6. Effects of EEB on Hepatic Antioxidant Enzymes Activity

 Several lines of evidence have established that excessive oxidative stress caused by reactive oxygen species (ROS) and reactive nitrogen species (RNS) can promote disease progression through oxidation of biomolecules such as DNA, lipids, and proteins [34, 35]. Oxidative stress contributes to diabetes and other human diseases [36]. Elevated levels of free fatty acid have been inhibiting insulin secretion by mitochondrial oxidation in the establishment of insulin resistance in diabetes mellitus [37]. An association between oxidative stress and insulin resistance has been reported in diabetes [38]. The results described above indicated that FRD induction resulted in lipid accumulation in the liver, resulting in insulin resistance. Therefore, the preventive effects of EEB and rutin/quercetin + rutin on oxidative stress in the liver of FRD-induced mice were evaluated in this study. The results suggested that EEB and rutin/quercetin + rutin administration significantly increased CAT, GR, GPx, and SOD activities in liver of FRD-induced C57BL/6 mice (Table 3).

## 4. Conclusion

In conclusion, we evaluated the effects of EEB, rutin, and quercetin on the insulin resistance pathway in FL83B hepatocytes with high-glucose-level-induced insulin resistance. We found that EEB, rutin, and quercetin may alleviate insulin resistance by improving insulin signaling via p-PKC activity inhibition and glucose uptake enhancement in insulin-resistant cells. In addition, we proposed that alleviation of insulin resistance was involved in the antioxidative effect of these phenolic acids in C57BL/6 mice induced by FRD (60%) for 8 weeks. We found that EEB and rutin/quercetin + rutin could improve hyperglycemia and hyperinsulinemia but quercetin administration did not show these activities, suggesting that rutin was major active compound of EEB. Taken together, the present study showed that EEB, rutin, and quercetin exerted antihyperglycemic and antioxidant activities because of their multiple effects, including antioxidative capacity and promotion of antioxidation enzymes, for protecting oxidative stress-induced insulin resistance in FL83B hepatocytes.

## Figures and Tables

**Table 1:** Effects of EEB on HOMA-IR by levels of blood glucose and insulin in FRD-induced rats.

Groups	Week 8		
	Glucose (mmol/L)	Insulin (mIU/L)	HOMA-IR
Normal	6.4 ± 0.4	10.3 ± 0.4	2.9 ± 0.4
HFD	7.8 ± 0.0^#^	18.1 ± 0.5^#^	6.3 ± 0.7^#^
HFD + quercetin	7.5 ± 0.1^#^	17.1 ± 0.7^#^	5.7 ± 0.2^#^
HFD + rutin	7.2 ± 0.1^#∗^	15.3 ± 0.4^#∗^	4.9 ± 0.4^#∗^
HFD + EEB	6.9 ± 0.4^#∗^	11.2 ± 0.3*	3.4 ± 0.8^#∗^
HFD + quercetin + rutin	7.0 ± 0.3^#∗^	13.6 ± 0.2*	4.2 ± 0.1^#∗^

Hyperglycemia and hyperinsulinemia in mice were induced by fructose-rich diet (FRD; 60%) for 8 weeks. The animals were randomly divided into 6 groups (*n* = 12), including (a) control, (b) fructose-rich diet (FRD), (c) FRD + quercetin (3 mg/kg bw), (d) FRD + rutin (11.5 mg/kg bw), (e) FRD + EEB (50 mg/kg bw), and (f) FRD + rutin + quercetin. The doses of rutin and quercetin were equivalent to those administered to the EEB administration group. Data are presented as the mean ± SEM. ^#^Significant difference from the control group (*P* < 0.05).  *Significant difference form the FRD group (*P* < 0.05). The statistics were shown by the *t*-test.

**Table 2:** Effect of EEB on hepatic and serum TC, TG, HDL, and LDL levels in FRD-induced rats at week 8.

Groups	Hepatic TG	Hepatic TC	Hepatic HDL-C	Hepatic LDL-C
		Concentration (mg/g)		
Normal	298.2 ± 3.6	26.6 ± 1.6	17.9 ± 0.2	373.4 ± 1.2
FRD	385.2 ± 7.1^#^	39.5 ± 1.3^#^	17.7 ± 0.8	457.2 ± 3.9^#^
FRD + quercetin	359.8 ± 4.2^#∗^	38.9 ± 2.6^#^	16.0 ± 0.3	429.7 ± 4.6^#^
FRD + rutin	322.2 ± 7.4^#∗^	29.6 ± 1.9^#∗^	17.1 ± 0.4	393.1 ± 3.4^#∗^
FRD + EEB	310.0 ± 6.2*	24.7 ± 1.4*	15.7 ± 0.4*	378.5 ± 2.0*
FRD + quercetin + rutin	317.9 ± 7.3*	27.9 ± 1.1*	16.7 ± 0.1	359.8 ± 10.1*

Groups	Plasma TG	Plasma TC	Plasma HDL-C	Plasma LDL-C
		Concentration (mg/dL)		

Normal	124.6±1.7	69.0 ± 1.1	71.8 ± 2.5	126.5 ± 1.5
FRD	238.0 ± 1.1*	94.3 ± 3.1^#^	52.6 ± 1.6^#^	239.5 ± 4.7^#^
FRD + quercetin	193.5 ± 6.6^#∗^	89.0 ± 5.3^#^	54.1 ± 3.7^#^	235.5 ± 5.6^#^
FRD + rutin	172.0 ± 3.3^#∗^	82.7 ± 2.8^#∗^	52.5 ± 5.3^#^	211.4 ± 1.6^#∗^
FRD + EEB	148.5 ± 8.7^#∗^	76.6 ± 2.4^#∗^	62.8 ± 2.2^#∗^	187.5 ± 3.9^#∗^
FRD + quercetin + rutin	138.2 ± 6.4*	80.5 ± 2.4^#∗^	59.4 ± 1.5^#∗^	193.2 ± 1.8^#∗^

Hyperglycemia and hyperinsulinmia in mice were induced by fructose-rich diet (FRD; 60%) for 8 weeks. The animals were randomly divided into 6 groups (*n* = 12), including (a) control, (b) fructose-rich diet (FRD), (c) FRD + quercetin (3 mg/kg bw), (d) FRD + rutin (11.5 mg/kg bw), (e) FRD + EEB (50 mg/kg bw), and (f) FRD + rutin + quercetin. The doses of rutin and quercetin were equivalent to those administered to the EEB administration group. Data are presented as the mean ± SEM. ^#^Significant difference from the control group (*P* < 0.05). *Significant difference form the FRD group (*P* < 0.05). The statistics were shown by the *t*-test.

**Table 3:** Effects of EEB on hepatic antioxidase activity in FRD-induced rats at week 8.

Groups	Hepatic antioxidant enzyme activity			
	CAT	GR	GPx	SOD
	nmol H_2_O_2_/min/mg protein	nmol NADPH/min/mg protein		U/mg protein
Normal	126.3 ± 1.6	5963 ± 11	5438 ± 32	65.8 ± 0.7
FRD	90.4 ± 3.0^#^	4543 ± 56^#^	4019 ± 57^#^	50.3 ± 0.6^#^
FRD + quercetin	120.8 ± 1.8*	4326 ± 52^#^	4089 ± 34^#^	58.5 ± 1.9^#∗^
FRD + rutin	117.9 ± 2.9*	5107 ± 60^#∗^	4101 ± 55^#^	65.0 ± 2.6*
FRD + EEB	127.1 ± 3.7*	6211 ± 58*	4739 ± 38^#∗^	67.1 ± 1.4*
FRD + quercetin + rutin	121.0 ± 2.0*	5842 ± 32*	4997 ± 22^#∗^	62.2 ± 1.7*

Hyperglycemia and hyperinsulinemia in mice were induced by fructose-rich diet (FRD; 60%) for 8 weeks. The animals were randomly divided into 6 groups (*n* = 12), including (a) control, (b) fructose-rich diet (FRD), (c) FRD + quercetin (3 mg/kg bw), (d) FRD + rutin (11.5 mg/kg bw), (e) FRD + EEB (50 mg/kg bw), and (f) FRD + rutin + quercetin. The doses of rutin and quercetin were equivalent to those administered to the EEB administration group. Data are presented as the mean ± SEM. ^#^Significant difference from the control group (*P* < 0.05). *Significant difference form the FRD group (*P* < 0.05). The statistics were shown by the *t*-test.

## References

[1] Jang EH, Ko JH, Ahn CW (2010). In vivo and in vitro application of black soybean peptides in the amelioration of endoplasmic reticulum stress and improvement of insulin resistance. *Life Sciences*.

[2] Zimmet P, Alberti KGMM, Shaw J (2001). Global and societal implications of the diabetes epidemic. *Nature*.

[3] Nagai Y, Nishio Y, Nakamura T, Maegawa H, Kikkawa R, Kashiwagi A (2002). Amelioration of high fructose-induced metabolic derangements by activation of PPAR*α*. *American Journal of Physiology*.

[4] Nagata R, Nishio Y, Sekine O (2004). Single nucleotide polymorphism (-468 Gly to Ala) at the promoter region of sterol regulatory element-binding protein-1c associates with genetic defect of fructose-induced hepatic lipogenesis. *Journal of Biological Chemistry*.

[5] Dekker MJ, Su Q, Baker C, Rutledge AC, Adeli K (2010). Fructose: a highly lipogenic nutrient implicated in insulin resistance, hepatic steatosis, and the metabolic syndrome. *American Journal of Physiology*.

[6] Adams SH, Stanhope KL, Grant RW, Cummings BP, Havel PJ (2008). Metabolic and endocrine profiles in response to systemic infusion of fructose and glucose in rhesus macaques. *Endocrinology*.

[7] Vos MB, McClain CJ (2009). Fructose takes a toll. *Hepatology*.

[8] Faeh D, Minehira K, Schwarz JM, Periasami R, Seongsu P, Tappy L (2005). Effect of fructose overfeeding and fish oil administration on hepatic de novo lipogenesis and insulin sensitivity in healthy men. *Diabetes*.

[9] Holasova M, Fiedlerova V, Smrcinova H, Orsak M, Lachman J, Vavreinova S (2002). Buckwheat—the source of antioxidant activity in functional foods. *Food Research International*.

[10] Liu CL, Chen YS, Yang JH, Chiang BH (2008). Antioxidant activity of tartary (*Fagopyrum tataricum* (L.) gaertn.) and common (*Fagopyrum esculentum* moench) buckwheat sprouts. *Journal of Agricultural and Food Chemistry*.

[11] Lee B-H, Hsu W-H, Pan T-M (2011). Inhibitory effects of dioscorea polysaccharide on TNF-*α*-induced insulin resistance in mouse FL83B cells. *Journal of Agricultural and Food Chemistry*.

[12] Schieber A, Keller P, Carle R (2001). Determination of phenolic acids and flavonoids of apple and pear by high-performance liquid chromatography. *Journal of Chromatography A*.

[13] Liu TP, Liu IM, Cheng JT (2005). Improvement of insulin resistance by Panax ginseng in fructose-rich chow-fed rats. *Hormone and Metabolic Research*.

[14] Sharma AK, Srinivasan BP (2009). Triple verses glimepiride plus metformin therapy on cardiovascular risk biomarkers and diabetic cardiomyopathy in insulin resistance type 2 diabetes mellitus rats. *European Journal of Pharmaceutical Sciences*.

[15] Matthews DR, Hosker JP, Rudenski AS (1985). Homeostasis model assessment: insulin resistance and *β*-cell function from fasting plasma glucose and insulin concentrations in man. *Diabetologia*.

[16] Jalal R, Bagheri SM, Moghimi A, Rasuli MB (2007). Hypoglycemic effect of aqueous shallot and garlic extracts in rats with fructose-induced insulin resistance. *Journal of Clinical Biochemistry and Nutrition*.

[17] Mohandas J, Marshall JJ, Duggin GG (1984). Low activities of glutathione-related enzymes as factors in the genesis of urinary bladder cancer. *Cancer Research*.

[18] Bellomo G, Mirabelli F, DiMonte D (1987). Formation and reduction of glutathione-protein mixed disulfides during oxidative stress. A study with isolated hepatocytes and menadione (2-methyl-1,4-naphthoquinone). *Biochemical Pharmacology*.

[19] Aebi H (1984). Catalase in vitro. *Methods in Enzymology*.

[20] Marklund S, Marklund G (1974). Involvement of the superoxide anion radical in the autoxidation of pyrogallol and a convenient assay for superoxide dismutase. *European Journal of Biochemistry*.

[21] Lee BH, Ho BY, Wang CT, Pan TM (2009). Red mold rice promoted antioxidase activity against oxidative injury and improved the memory ability of zinc-deficient rats. *Journal of Agricultural and Food Chemistry*.

[22] Gray P (1964). *Handbook of Basic Microtechnique*.

[23] Alessi DR, Cohen P (1998). Mechanism of activation and function of protein kinase B. *Current Opinion in Genetics and Development*.

[24] Kola B, Boscaro M, Rutter GA, Grossman AB, Korbonits M (2006). Expanding role of AMPK in endocrinology. *Trends in Endocrinology and Metabolism*.

[25] Kahn BB, Alquier T, Carling D, Hardie DG (2005). AMP-activated protein kinase: ancient energy gauge provides clues to modern understanding of metabolism. *Cell Metabolism*.

[26] Lee B-H, Hsu W-H, Liao T-H, Pan T-M (2011). The Monascus metabolite monascin against TNF-*α*-induced insulin resistance via suppressing PPAR-*γ* phosphorylation in C2C12 myotubes. *Food and Chemical Toxicology*.

[27] Jialal I, Venugopal SK (2002). Oxidative strees, inflammation, and diabetic vasculopathies: the role of alpha tocopherol therapy. *Free Radical Research*.

[28] Jain SK, Kannan K, Lim G, Matthews-Greek J, McVie R, Bocchini JA (2003). Elevated blood interleukin-6 levels in hyperketonemic type 1 diabetic patients and secretion by acetoacetate-treated cultured U937 monocytes. *Diabetes Care*.

[29] Shanmugam N, Reddy MA, Guha M, Natarajan R (2003). High glucose-induced expression of proinflammatory cytokine and chemokine genes in monocytic cells. *Diabetes*.

[30] Feige JN, Gelman L, Michalik L, Desvergne B, Wahli W (2006). From molecular action to physiological outputs: peroxisome proliferator-activated receptors are nuclear receptors at the crossroads of key cellular functions. *Progress in Lipid Research*.

[31] Díaz-Delfín J, Morales M, Caelles C (2007). Hypoglycemic action of thiazolidinediones/peroxisome proliferator-activated receptor *γ* by inhibition of the c-Jun NH2-terminal kinase pathway. *Diabetes*.

[32] Dekker MJ, Su Q, Baker C, Rutledge AC, Adeli K (2010). Fructose: a highly lipogenic nutrient implicated in insulin resistance, hepatic steatosis, and the metabolic syndrome. *American Journal of Physiology*.

[33] Salgin B, Marcovecchio ML, Williams RM (2009). Effects of growth hormone and free fatty acids on insulin sensitivity in patients with type 1 diabetes. *Journal of Clinical Endocrinology and Metabolism*.

[34] Burke A, FitzGerald GA (2003). Oxidative stress and smoking-induced vascular injury. *Progress in Cardiovascular Diseases*.

[35] Nakao LS, Iwai LK, Kalil J, Augusto O (2003). Radical production from free and peptide-bound methionine sulfoxide oxidation by peroxynitrite and hydrogen peroxide/iron(II). *FEBS Letters*.

[36] Niedowicz DM, Daleke DL (2005). The role of oxidative stress in diabetic complications. *Cell Biochemistry and Biophysics*.

[37] Shah A, Mehta N, Reilly MP (2008). Adipose inflammation, insulin resistance, and cardiovascular disease. *Journal of Parenteral and Enteral Nutrition*.

[38] Videla LA (2009). Oxidative stress signaling underlying liver disease and hepatoprotective mechanisms. *World Journal of Hepatology*.

